# Effectiveness of a structured diabetes self-management education program using high-definition video technology in type 1 diabetes: an interventional study

**DOI:** 10.3389/fendo.2026.1812520

**Published:** 2026-06-17

**Authors:** Mohamed Hosny Nagla, Mostafa A. Khalifa, Marwa Farouk Mira, Tarek Mohamed Faird, Ahmed Adel Mohamed, Abdullah F. Ghrabat, Wafaa Khadr, Iyad Basher Alsarhi, Abeer Atef El Ashmawy

**Affiliations:** 1 Pediatric Intensive Care Unit, Al Salama Hospital, Jeddah, Saudi Arabia; 2 Faculty of Medicine, Cairo University, Cairo, Egypt; 3 Pediatrics Faculty of Medicine, Cairo University, Cairo, Egypt; 4 Child Health Department, National Research Centre, Cairo, Egypt; 5 Faculty of Medicine, Zagazig University, Zagazig, Egypt; 6 College of Medicine, University of Baghdad, Baghdad, Iraq; 7 Primary Health Care Department, Ministry of Health and Population, Cairo, Egypt

**Keywords:** diabetes management, glycemic control, pediatric endocrinology, self-management education, type 1 diabetes

## Abstract

**Objective:**

One of the most common chronic disorders in children is type 1 diabetes (T1D). It requires regular evaluation, stringent glycemic control, and comprehensive education on disease management. The aim of this work is to evaluate the effectiveness of Audio-visual technology as a tool for self-management education in pediatric patients with T1D attending diabetes outpatient clinics in Health Insurance Hospitals.

**Patients and methods:**

his interventional pre–post study was conducted on 100 patients with T1D at the Diabetes Endocrine Metabolic Pediatric Unit (DEMPU) of Cairo University Children’s Hospital and the Diabetes Outpatient Clinic of 6th October Hospital for Health Insurance, Egypt, between May 2016 and February 2017. Participants were consecutively recruited from eligible patients attending routine outpatient clinic visits during the study period. A structured questionnaire assessing diabetes self-management knowledge was administered at baseline, immediately post-intervention, and at 2- and 4-month follow-up after a video-based educational program.

**Results:**

Diabetes-related knowledge scores significantly improved from 36.36 ± 7.94 at baseline to 42.07 ± 7.82 post-intervention, with sustained retention at 4 months (42.61 ± 8.47, p< 0.001). HbA1c levels significantly decreased at 4 months (mean change −0.81 ± 1.18%), with 78% of participants showing improvement (p< 0.001). The proportion achieving target glycemic control increased from 19% to 29%, while poor control decreased from 41% to 30%. Knowledge gain was independently associated with HbA1c reduction.

**Conclusion:**

A structured diabetes self-management education program using high-definition video technology was associated with significant improvements in diabetes knowledge and glycemic control. These findings support the role of structured educational interventions as an effective and scalable strategy for improving clinical outcomes in patients with type 1 diabetes.

**Clinical Relevance:**

This study highlights the utility of high-definition video technology as an effective educational tool to improve diabetes knowledge and self-management in pediatric patients with T1D, offering a scalable solution for diabetes education programs.

## 1 Introduction

Diabetes is a chronic metabolic disease characterized by hyperglycemia, arising from an imbalance in insulin secretion or impaired responsiveness of target tissues. Based on etiopathogenesis, diabetes is categorized into type 1 and type 2. Type 1 diabetes (T1D) is an autoimmune condition marked by the destruction of beta cells in the islets of Langerhans, leading to reduced endogenous insulin production (1). Insufficient insulin levels and impaired tissue responses disrupt the metabolism of carbohydrates, proteins, and fats, resulting in metabolic abnormalities. Diagnosis of diabetes relies on blood glucose level (BGL) assessments, including hemoglobin A1C (HbA1c), fasting plasma glucose (FPG), and the 2-hour plasma glucose (2-h PG) during an oral glucose tolerance test (OGTT) (2).

According to the International Diabetes Federation (IDF) and the World Health Organization (WHO), in 2022, there were approximately 1.52 million children and adolescents with T1D among the 8.75 million patients globally (3).

Among the critical components of diabetes management, patient education plays a pivotal role. For children and young individuals under 20 years of age, who are primarily affected by T1D, robust education programs are essential for fostering effective disease management (4). Evidence suggests that educational interventions improve HbA1c levels, particularly during the first year post-diagnosis (5).

Despite the well-established importance of diabetes self-management education, limited evidence exists regarding the effectiveness of structured audio-visual educational tools in improving glycemic control among children with T1D, particularly in low- and middle-income countries.

Audio-visual educational interventions may be especially effective in pediatric populations, as they enhance engagement, improve information retention, and help overcome literacy-related barriers. Moreover, such approaches are highly practical in resource-limited settings where access to continuous face-to-face education may be constrained.

Developing countries bear a significant burden of the disease, with projections indicating a doubling of cases in the Middle East and North Africa (MENA) region by 2045 (6). Egypt, one of the MENA countries, ranks among the top nations globally in terms of diabetes prevalence (7, 8).

Therefore, this study aimed to evaluate the effectiveness of a structured diabetes self-management education program using high-definition video technology in improving diabetes-related knowledge among children with T1D, as well as its potential impact on glycemic control (HbA1c).

## 2 Methods

### 2.1 Study design and setting

This study was designed as an interventional pre–post study and was conducted at the Diabetes Endocrine Metabolic Pediatric Unit (DEMPU) of Cairo University Children’s Hospital and the Diabetes Outpatient Clinic of 6th October Hospital for Health Insurance, Egypt. The study was carried out over a 10-month period from May 2016 to February 2017.

### 2.2 Study population and eligibility criteria

The study population consisted of children and adolescents diagnosed with T1D who attended routine follow-up visits at the participating outpatient clinics during the study period. Eligible participants were those aged 18 years or younger with a confirmed diagnosis of T1D who had not previously received any structured diabetes self-management education.

Patients were excluded if they had a diagnosis of type 2 diabetes mellitus, were 19 years of age or older, or had previously participated in a formal diabetes self-management education program.

### 2.3 Sample size and recruitment

A total of 100 children and adolescents with T1D were recruited consecutively during routine outpatient clinic visits. Participants were consecutively recruited from eligible patients attending routine outpatient clinic visits during the study period. No randomization was performed, as this was a single-arm pre–post interventional study without a control group. Written informed consent was obtained from parents or legal guardians prior to participation.

**Figure 1:**
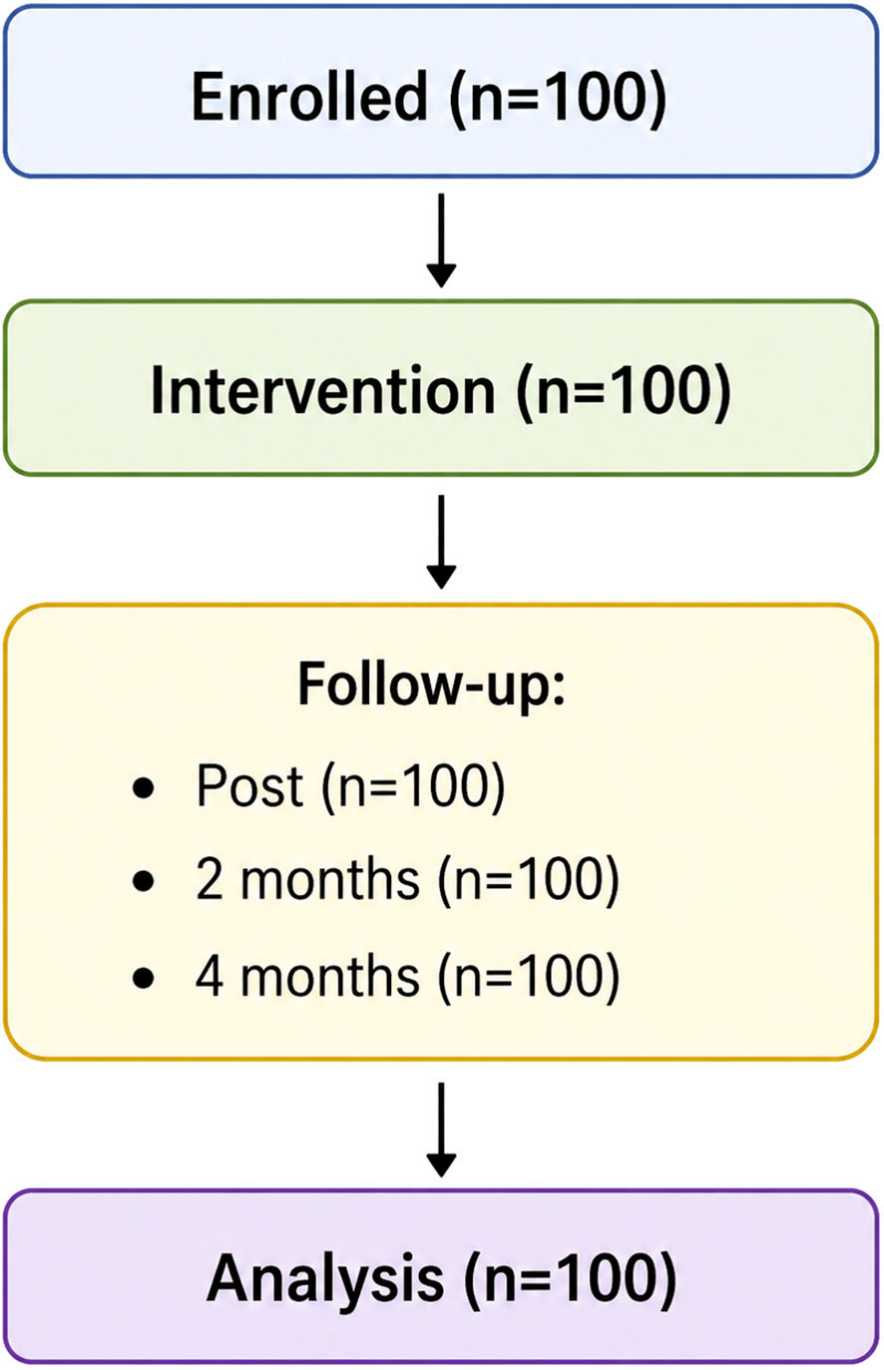
Participant flow diagram.

A flow diagram illustrating participant enrollment, intervention, follow-up, and analysis was constructed to enhance reporting transparency (**Figure 1****).**

### 2.4 Ethical consideration

The study was conducted in accordance with the Declaration of Helsinki. Ethical approval was obtained from the institutional ethics committee. Written informed consent was obtained from the parents or legal guardians of all participants prior to enrollment.

### 2.5 Educational intervention

All participants received a structured diabetes self-management education program delivered through high-definition audio-visual technology. The educational intervention was provided as a single session and was tailored to each patient’s age, developmental stage, lifestyle, and cultural background. The program was developed in accordance with international recommendations for pediatric diabetes education and the principles of diabetes self-management education programs, and was designed to enhance participants’ understanding of diabetes self-management while promoting engagement and motivation (9).

The intervention consisted of a 45-minute high-definition educational video. The first part of the video provided an overview of T1D, including its etiology, common clinical manifestations, and basic principles of blood glucose monitoring, with a demonstration of proper glucometer use. The second part focused on insulin therapy, addressing different types of insulin, appropriate injection sites, correct injection techniques, and the proper use of insulin pens. The third part covered essential nutritional principles for children with diabetes, with particular emphasis on carbohydrate intake and meal planning.

Following the educational video, individualized counseling was provided by a trained dietitian. Daily energy and carbohydrate requirements were calculated for each participant based on age, body weight, and body mass index. Carbohydrate counting was explained using standard exchange concepts, and dietary recommendations were individualized according to insulin regimen, meal frequency, and daily activity levels.

The final component of the intervention consisted of a motivational audio-visual segment portraying a child with T1D leading an active and healthy life while adhering to recommended diabetes management practices. This segment aimed to reinforce positive behavioral changes, encourage adherence to treatment, and increase awareness of the prevention and recognition of acute complications such as hypoglycemia and hyperglycemia. Parents and caregivers were encouraged to participate in the session to support ongoing diabetes management at home.

### 2.6 Outcome measures

The outcome measures were predefined to assess the effectiveness of the video-based diabetes self-management education program.

The primary outcome of this study was improvement in diabetes-related knowledge assessed using the structured questionnaire. The secondary outcome was improvement in glycemic control measured by glycated hemoglobin (HbA1c).

Glycemic control was evaluated using glycated hemoglobin (HbA1c), measured at baseline before the educational intervention and repeated four months after program completion. Overall diabetes-related knowledge was assessed using a structured questionnaire administered before the intervention and during follow-up.

The secondary outcomes were improvements in specific domains of diabetes self-management knowledge, including the identification and management of hypoglycemia and hyperglycemia, insulin administration techniques, dietary carbohydrate management, and awareness of psychological support and the importance of regular follow-up. These knowledge domains consisted of the following number of items: general knowledge about diabetes (20 items), hypoglycemia and hyperglycemia management (20 items), injection technique (7 items), dietary carbohydrate distribution (15 items), and psychological support and awareness of follow-up (9 items).

Outcome measures were selected to provide clinically relevant indicators of improved diabetes self-management and to align with the predefined objectives of the study.

### 2.7 Data collection procedures

Data were collected using a structured Arabic multiple-choice questionnaire designed to assess diabetes self-management knowledge. The questionnaire was administered to participants and/or their parents both before and after the educational intervention. It was administered in written form for educated participants and orally for uneducated participants; however, most parents preferred oral administration regardless of educational level. In cases where the child was too young to respond independently, the questionnaire was completed by the parents or caregivers on behalf of the child. Therefore, the responses were considered representative of the patient–family unit rather than being analyzed separately for parents and children.

Each question was assigned a score of +1 for a correct answer and 0 for an incorrect answer. Put right-or-wrong questions were scored according to the number of correct choices selected. Domain-specific scores were calculated by summing the correct responses within each domain. For example, hypoglycemia knowledge was assessed using twenty questions; if the patient or their parent correctly answered four out of twenty questions, the corresponding score was calculated as 4/20.

The same questionnaire was re-administered during follow-up assessments to evaluate changes in knowledge and retention over time. The assessment tools used, the evaluated knowledge domains, scoring systems, and timing of assessments are summarized in **Table 1**.

**Table 1:** Assessment tools and outcome measures used in the study.

Assessment tool	Domain/component	Number of items	Scoring method	Timing of assessment
Diabetes Self-Management Knowledge Questionnaire	General knowledge about diabetes	20	One point for each correct answer, 0 for incorrect	Baseline, immediately post-intervention, 2 months, 4 months
	Hypoglycemia and hyperglycemia management	20	One point for each correct answer, 0 for incorrect	Baseline, immediately post-intervention, 2 months, 4 months
	Insulin injection technique	7	One point for each correct answer, 0 for incorrect	Baseline, immediately post-intervention, 2 months, 4 months
	Dietary carbohydrate distribution	15	One point for each correct answer, 0 for incorrect	Baseline, immediately post-intervention, 2 months, 4 months
	Psychological support and follow-up awareness	9	One point for each correct answer, 0 for incorrect	Baseline, immediately post-intervention, 2 months, 4 months
Socioeconomic Status Scale	Education and cultural domain (parents)	30	Domain-based scoring	Baseline
	Occupational domain (parents)	10	Domain-based scoring	Baseline
	Family possession domain	12	One point for each available item	Baseline
	Family domain	10	Domain-based scoring	Baseline
	Home sanitation domain	12	Domain-based scoring	Baseline
	Economic domain	5	Domain-based scoring	Baseline
	Health care domain	5	Domain-based scoring	Baseline
Pediatric Quality of Life Inventory (PedsQL v4.0)	Physical functioning	8	Standardized PedsQL scoring	Baseline
	Emotional and interpersonal functioning	5	Standardized PedsQL scoring	Baseline
	School functioning	4	Standardized PedsQL scoring	Baseline

### 2.8 Reliability and internal consistency of the questionnaire

The internal consistency of the diabetes self-management knowledge questionnaire was assessed using Cronbach’s alpha coefficient across its domains. Test–retest reliability was evaluated using the intraclass correlation coefficient (ICC) based on repeated measurements over time. These analyses were performed to ensure the reliability and stability of the assessment tool.

### 2.9 Follow-up and study duration

Participants were followed up at 2 and 4 months after the educational intervention. Follow-up was conducted through telemedicine using phone calls and short message service (SMS). During follow-up, the same questionnaire was re-administered, and incorrect responses were clarified to reinforce learning.

Assessments were conducted at four time points: baseline (pre-intervention), immediately post-intervention, and at 2- and 4-month follow-up.

### 2.10 Statistical analysis

Statistical analysis was performed using Python 3, utilizing the SciPy, Statsmodels, Pandas, Matplotlib, and Seaborn libraries. The normality of continuous variables was assessed using the Shapiro-Wilk test. Due to the non-normal distribution of most continuous variables, non-parametric statistical methods were predominantly employed. Quantitative data were expressed as mean ± standard deviation (SD) along with median and interquartile range (IQR), while qualitative/categorical data were expressed as frequencies and percentages.

Between-group differences for continuous variables were evaluated using the Mann-Whitney U test for two groups and the Kruskal-Wallis H test for three or more groups. Longitudinal changes across the four time points were analyzed using the Friedman test, followed by *post-hoc* pairwise comparisons using the Wilcoxon signed-rank test. To control for multiple comparisons during *post-hoc* testing, a Bonferroni correction was applied, adjusting the significance threshold to *α* = 0.0083. Categorical variables were compared using Pearson’s Chi-square or Fisher’s Exact tests, where appropriate.

Associations between variables were evaluated using Spearman’s rank correlation (*ρ*). To identify independent predictors of clinical outcomes (e.g., HbA1c reduction and complications), Ordinary Least Squares (OLS) multiple linear regression and logistic regression models were constructed. Effect sizes were calculated where applicable (e.g., rank-biserial correlation [*r*], Cohen’s *d*, and epsilon-squared $\left[\eta_{\varepsilon }^{2}\right]$). Unless otherwise specified by Bonferroni adjustments, a two-tailed *p*-value ≤ 0.05 was considered statistically significant, a *p*-value< 0.001 was considered highly significant, and a *p*-value > 0.05 was considered insignificant.

### 2.11 Assessments

#### 2.11.1 Clinical and laboratory assessment

A complete clinical history was obtained for all participants, including age at onset of diabetes, calculated duration of disease, parental educational level, socioeconomic status, quality of life, and type and regimen of insulin therapy. A comprehensive systemic examination was performed to identify any diabetes-related complications.

Body weight was measured using calibrated weighing scales according to standardized procedures appropriate for the child’s age and condition. Injection sites were examined to detect any local complications related to insulin administration.

Glycemic control was assessed using glycated hemoglobin (HbA1c%), which was measured at baseline and repeated at the 4-month follow-up. HbA1c was measured in the central laboratories of DEMPU and 6th October Health Insurance Hospital using high-performance liquid chromatography (HPLC) on a Bio-Rad D-10™ system (NGSP-certified, intra-assay coefficient of variation< 2.0%). All measurements were performed according to standard clinical protocols, and the results were retrieved from the patients’ routine medical records. Glycemic control was classified in accordance with the recommendations of the American Diabetes Association (ADA) and the International Society for Pediatric and Adolescent Diabetes (ISPAD) clinical practice guidelines as good, moderate, or poor metabolic control based on established HbA1c% thresholds (10, 11).

A history of diabetic ketoacidosis was documented using standard biochemical criteria, and severe hypoglycemia was defined based on clinical presentation and the need for assisted or parenteral treatment.

Hospital admissions related to acute glycemic events (e.g., diabetic ketoacidosis and severe hypoglycemia) were also recorded at baseline and monitored during the follow-up period.

#### 2.11.2 Assessment of chronic diabetes complications

Chronic diabetes-related complications were assessed using standard clinical methods. Diabetic retinopathy was evaluated through ophthalmologic examination, diabetic nephropathy was assessed based on urinary protein excretion, and diabetic neuropathy was evaluated clinically, with electrophysiological studies performed when indicated. Complications were classified according to established clinical criteria.

#### 2.11.3 Socioeconomic status assessment

Socioeconomic status was evaluated using the validated El-Gilany et al. (2012) scale (12), which assesses multiple domains and provides a composite score for socioeconomic classification. Participants were categorized into extremely low, low, middle, or high socioeconomic levels based on calculated quartiles (**Table 1**).

#### 2.11.4 Quality of life assessment

Quality of life was assessed using the Pediatric Quality of Life Inventory (PedsQL), version 4.0 developed by Varni et al. (13). The instrument evaluates physical, emotional, and school functioning domains. Scores were calculated and interpreted according to standardized guidelines, with higher scores indicating better quality of life (**Table 1**).

Quality of life (QoL) was assessed only at baseline as part of the pre-intervention questionnaire and was not reassessed after the educational intervention. Accordingly, QoL was not considered a longitudinal outcome in this study.

## 3 Results

A total of 100 children and adolescents were enrolled during routine health maintenance visits between May 2016 and February 2017. All 100 participants (100%) completed the full 4-month follow-up period, with no attrition recorded.

Due to the non-normal distribution of most continuous variables (confirmed via Shapiro-Wilk testing), non-parametric statistics were employed throughout the analysis. Central tendencies are reported as Mean ± SD and Median (IQR). Between-group differences were assessed using Mann-Whitney U (MWU) tests for two groups and Kruskal-Wallis H tests for three or more groups. Longitudinal changes across the four time points were evaluated using the Friedman test, followed by *post-hoc* pairwise Wilcoxon signed-rank tests with a Bonferroni-corrected alpha of 0.0083. Categorical variables were compared using Chi-square or Fisher’s Exact tests, while associations were evaluated using Spearman’s rank correlation (ρ).

### 3.1 Demographics and clinical characteristics

**Figure 2:**
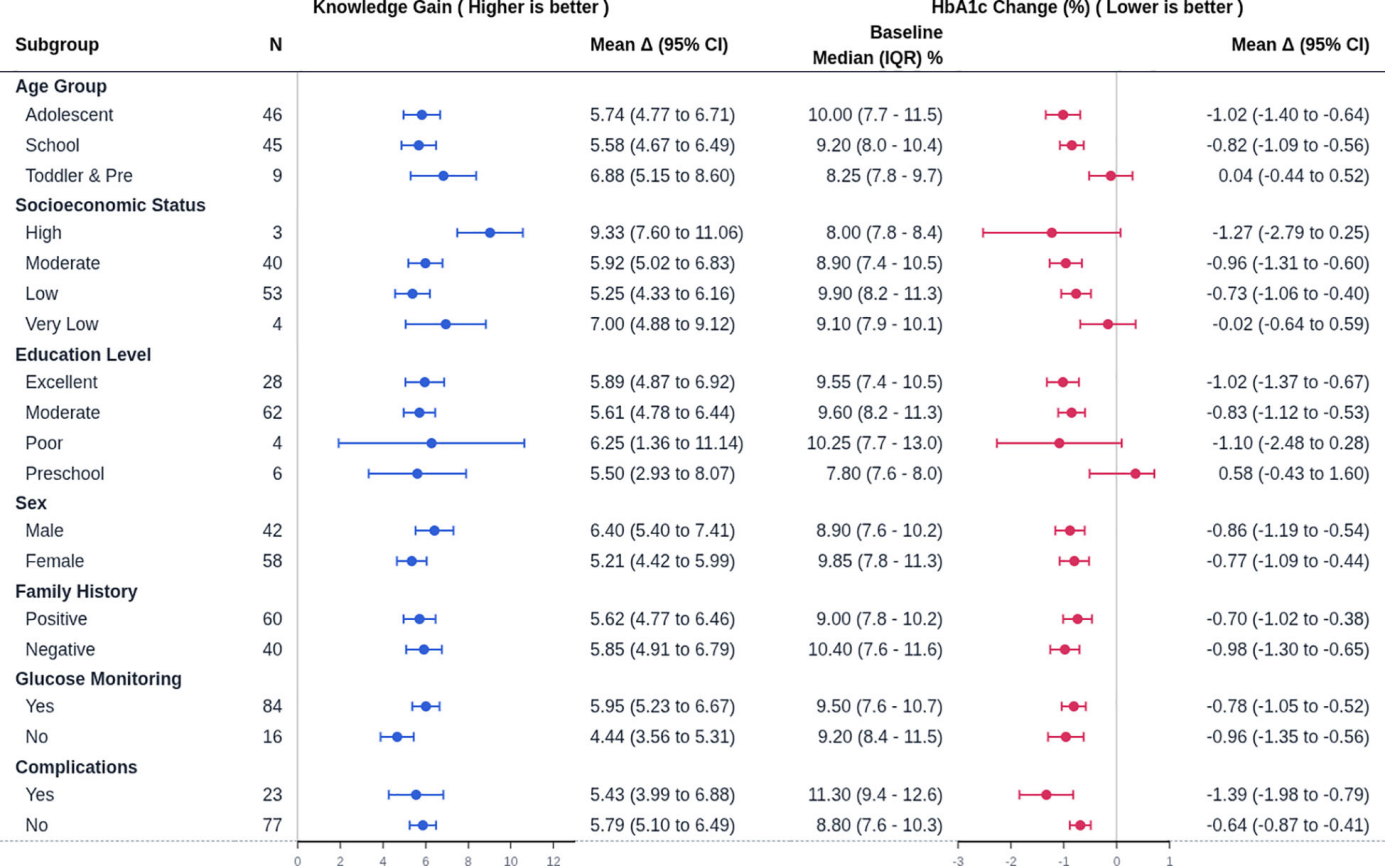
Population overview, with a comparison of knowledge and HbA1c after a 4-month questionnaire follow-up.

The cohort was predominantly female (58.0%) with a mean age of 11.51 ± 4.08 years (median = 12.0 years; IQR: 9–14). The majority of participants were of school age (45.0%) or adolescent (46.0%). The El-Gilany et al. socioeconomic classification (12) revealed that 57.0% of the cohort belonged to the low or very low SES categories. Subcutaneous insulin injection was the primary mode of administration (97.0%), with only one patient utilizing an insulin pump. Baseline demographic and clinical characteristics are shown in **Figure 2**.

At baseline, the mean duration of illness was 3.65 ± 3.13 years. Despite this relatively short mean disease duration, glycemic control was profoundly suboptimal. The median baseline HbA1c was 9.35 (7.70–11.20) %, with only 19.0% of patients meeting the American Diabetes Association (ADA) target of HbA1c ≤ 7.5%. The remaining patients exhibited fair (40.0%) or poor (41.0%, HbA1c ≥ 10%) metabolic control.

### 3.2 Primary outcome: improvement in diabetes knowledge

Baseline diabetes knowledge was assessed across five domains, with patients scoring a mean total of 36.36 ± 7.94 (51.2% of the maximum 71 points). The lowest baseline scores were observed in the “Follow-up & Psychological Support” (43.0% of maximum) and “Knowledge of Complications” (46.8% of maximum) domains.

Following the educational intervention, total knowledge scores significantly increased to 42.07 ± 7.82 (59.3% of maximum) immediately post-intervention. Repeated-measures analysis confirmed a highly significant longitudinal improvement across all time points (Friedman *χ*^2^ = 169.65, *p* < 0.001).

Pairwise comparisons (Bonferroni-corrected, *α* = 0.0083) revealed very large effect sizes for the gains from baseline to post-intervention. A maximal effect size was observed for the Total Score (*r* = 1.000, *p_adj_* < 0.001), with similarly large effects in the “Follow-up & Psychological Support” (*r* = 1.000, *p_adj_* < 0.001) and “Knowledge of Complications” (*r* = 0.991, *p_adj_* < 0.001) domains.

**Figure 3:**
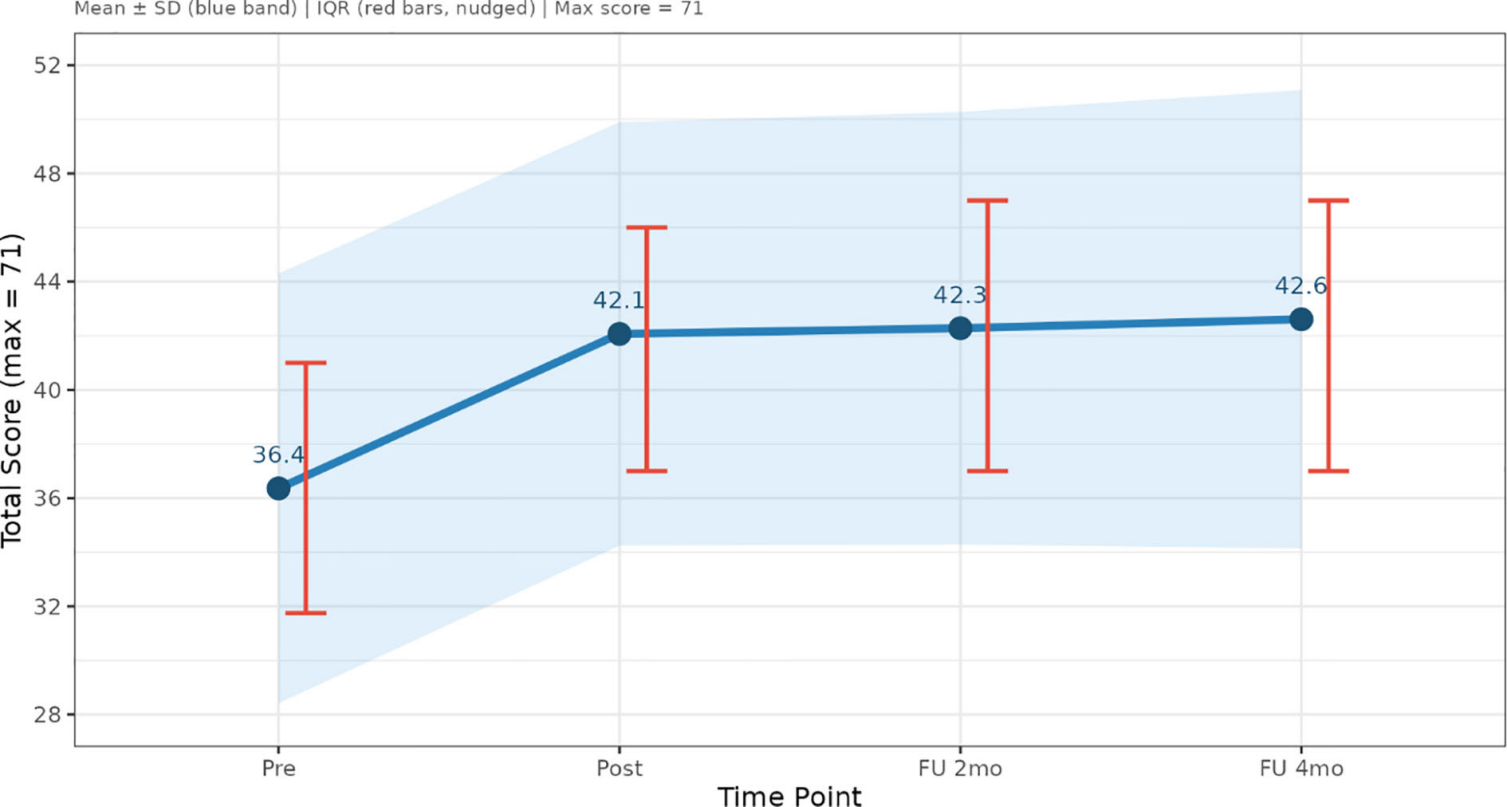
Total knowledge score trajectory across time points.

These knowledge gains were retained throughout the study period. Assessments at 2 and 4 months showed no significant score decay across any domain. Total scores at the 4-month follow-up (42.61 ± 8.47) did not significantly differ from the immediate post-intervention scores (Median Δ = +0.50 points; *p_adj_* = 0.738; *r* = 0.189), indicating sustained retention (**Figure 3**; **Table 2**).

**Table 2:** Longitudinal changes and effect sizes in diabetes knowledge scores.

Domain *(max score)*	Baseline (Pre) mean ± SD[median]	Post-intervention mean ± SD[median]	4-month follow-upmean ± SD[median]	Overall trend (Friedman *χ*^2^)	Immediate GainPre *vs* post *p_adj_ (Effect Size r)*	RetentionPost *vs* 4-Mo *p_adj_ (effect size r)*
General Knowledge *(20)*	11.63 ± 3.08[12.00]	13.02 ± 2.87[13.00]	13.26 ± 2.86[13.00]	122.69*p< 0.001*	*< 0.001 (0.962)*	0.648 *(0.212)*
Complications *(20)*	9.36 ± 3.11[9.00]	11.21 ± 2.79[11.00]	11.26 ± 3.02[11.00]	113.11*p< 0.001*	*< 0.001 (0.991)*	1.000 *(0.051)*
Insulin Injection *(7)*	3.95 ± 1.12[4.00]	4.50 ± 1.09[5.00]	4.58 ± 1.11[5.00]	56.41*p< 0.001*	*< 0.001 (0.861)*	1.000 *(0.166)*
Diabetic Nutrition *(15)*	7.55 ± 2.28[7.00]	8.74 ± 2.01[9.00]	8.80 ± 2.42[9.00]	89.40*p< 0.001*	*< 0.001 (0.885)*	1.000 *(0.185)*
Follow-up & Psych. *(9)*	3.87 ± 1.73[4.00]	4.60 ± 1.48[4.00]	4.71 ± 1.65[5.00]	73.97*p< 0.001*	*< 0.001 (1.000)*	1.000 *(0.109)*
TOTAL SCORE *(71)*	36.36 ± 7.94[36.00]	42.07 ± 7.82[42.00]	42.61 ± 8.47[42.50]	**169.65*p< 0.001*	*< 0.001 (1.000)*	0.738 *(0.189)*

### 3.3 Changes in glycemic control following the intervention

Following the self-management educational intervention, patients demonstrated a statistically significant and clinically meaningful improvement in glycemic control. Mean HbA1c difference was **−0.81 ± 1.18%**, moving median to 8.65 (7.30 - 10.3)% at the 4-month follow-up (Wilcoxon *W* = 690.0, *p* < 0.001, Cohen’s *d* ≈ 0.423).

Overall, 78.0% of the cohort achieved a reduction in HbA1c. The proportion of patients achieving the ADA target of good metabolic control (HbA1c ≤ 7.5%) increased from 19.0% to 29.0%, while the proportion of patients in the poor control category (HbA1c ≥ 10%) decreased from 41.0% to 30.0% (**Table 3**). Analysis of categorical shifts revealed that while 14 patients improved from fair to good control, 26 of the 41 initially poorly controlled patients remained in the poor category post-intervention, indicating a persistent high-risk subgroup.

**Table 3:** Metabolic control category: baseline *vs* post.

Metabolic control category	Baseline	Post (4 mo)
Good (≤7.5%)	19	29
Fair (7.6–9.9%)	40	41
Poor (≥10%)	41	30

### 3.4 Predictors of glycemic improvement

**Figure 4:**
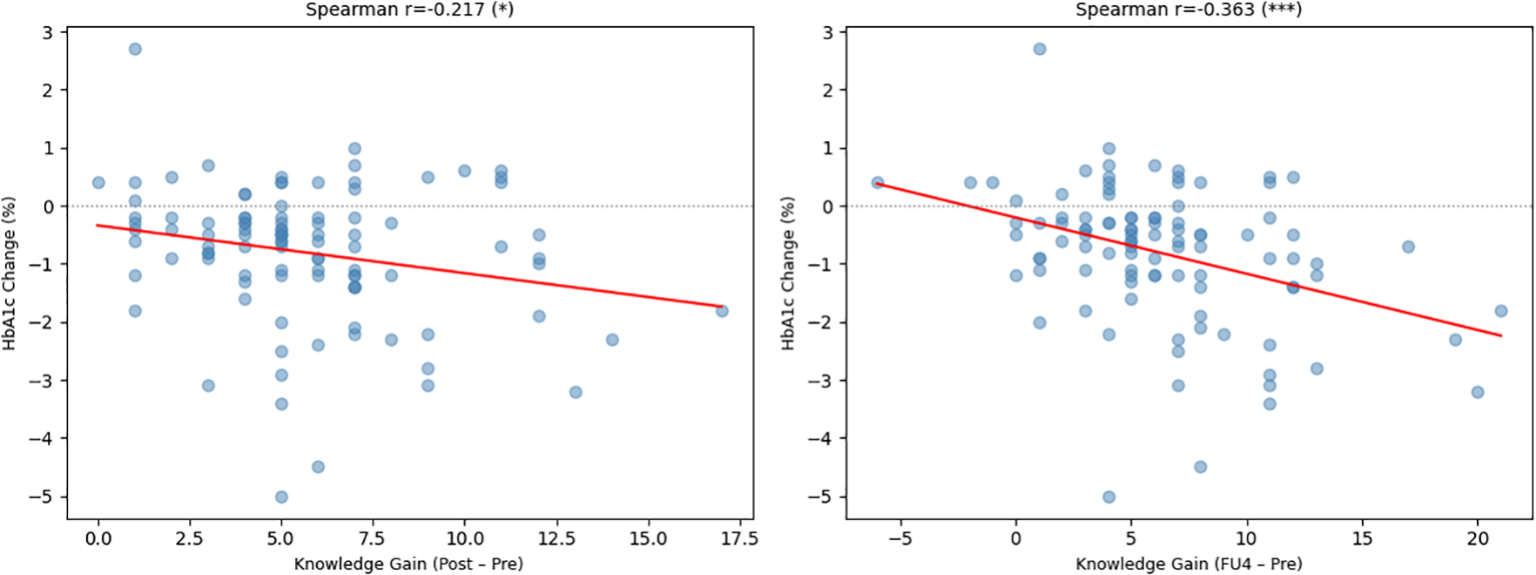
HbA1c change *vs* knowledge score gain.

To elucidate the mechanisms driving HbA1c reduction, correlation and multiple linear regression analyses were performed. A significant inverse correlation was identified between the magnitude of knowledge gain and HbA1c change (Spearman *ρ* = −0.322, *p* = 0.001 at post-intervention; *ρ* = −0.363, *p* < 0.001 at 4-month follow-up), establishing a clear mechanistic link between educational attainment and glycemic improvement (**Figure 4**).

In a multivariate OLS regression model adjusting for sex, age, disease duration, baseline HbA1c, SES, education, monitoring, and baseline knowledge, post-intervention knowledge gain independently predicted HbA1c reduction (*β* = −0.125, *p* < 0.001). The overall model was statistically significant (*F*(9,90) = 7.528, *p* < 0.001) and explained 42.9% of the variance in HbA1c change (Adjusted *R*^2^ = 0.372) (**Table 4**).

**Table 4:** OLS multiple regression analysis of predictors for HbA1c change.

Predictor	β Coefficient	Standard error (SE)	95% confidence interval	*t*-value	*p*-value
Constant	5.236	0.848	[3.552, 6.921]	6.176	< 0.001
Sex	-0.128	0.204	[-0.534, 0.279]	-0.624	0.534
Age	-0.053	0.032	[-0.116, 0.010]	-1.669	0.099
Disease Duration	+0.044	0.037	[-0.030, 0.118]	1.188	0.238
Baseline HbA1c	-0.295	0.051	[-0.396, -0.193]	-5.786	< 0.001
SES	+0.003	0.010	[-0.018, 0.024]	0.295	0.769
Education	-0.129	0.154	[-0.436, 0.177]	-0.837	0.405
Glucose Monitoring	+0.193	0.266	[-0.336, 0.721]	0.724	0.471
Knowledge Gain	-0.125	0.034	[-0.193, -0.057]	-3.638	< 0.001
Pre-Intervention Knowledge	-0.056	0.016	[-0.087, -0.025]	-3.610	0.001

Baseline HbA1c strongly predicted reduction (*β* = −0.295, *p* < 0.001) within a statistically valid model (Durbin-Watson = 2.21; Jarque-Bera *p* = 0.085).

### 3.5 Subgroup analyses

#### 3.5.1 HbA1c change by subgroup

Significant within-group reductions in HbA1c were observed across most subgroups, exhibiting large effect sizes (*r* > 0.50) among males, females, school-aged children, adolescents, and those with fair or poor baseline metabolic control (**Figure 2**). Notably, the intervention did not yield significant HbA1c improvements in the toddler/preschool age group (*p* = 1.000) or among those in the lowest socioeconomic and education brackets.

Between-group comparisons revealed that intervention response was significantly moderated by Age Group (*p* = 0.033), Baseline HbA1c Category (*p* = 0.002), and Education Level (*p* = 0.024). The greatest absolute HbA1c reductions were observed in patients with poor baseline metabolic control (Median Δ = -0.90%), adolescents (Median Δ = -0.70%), and those whose guardians had excellent education (Median Δ = -0.70%). Sex, Family History, Glucose Monitoring, and Socioeconomic Status (SES) did not significantly differentiate HbA1c responses between groups (**Table 5**).

**Table 5:** HbA1c change by subgroup.

*Subgroup*	*Category*	*N*	*Median Δ HbA1c [IQR]*	*Within-group p-value (r)*	*Between-group p-value (ES)*
*Sex*	Male	42	-0.55 [-1.00, -0.35]	< 0.001 (*r* = 0.71)	0.698 (*r_rb_* = 0.05)
	Female	58	-0.60 [-0.90, -0.40]	< 0.001 (*r* = 0.57)	
*Age Group*	Toddler/Pre (≤yr)	9	+0.30 [-0.70, +0.70]	1.000 (*r* = 0.00)	0.033 ( $\eta_{\varepsilon }^{2}=0.05$)
	School (6–12yr)	45	-0.60 [-0.90, -0.40]	< 0.001 (*r* = 0.74)	
	Adolescent (13–18yr)	46	-0.70 [-1.05, -0.50]	< 0.001 (*r* = 0.68)	
*Baseline HbA1c*	Good (≤7.5%)	19	-0.30 [-0.50, +0.40]	0.285 (*r* = 0.25)	0.002 ( $\eta_{\varepsilon }^{2}=0.11$)
	Fair (7.6–9.9%)	40	-0.60 [-0.85, -0.20]	< 0.001 (*r* = 0.54)	
	Poor (≥10%)	41	-0.90 [-1.90, -0.50]	< 0.001 (*r* = 0.81)	
*SES*	Very Low	4	0.00 [-0.70, +0.60]	1.000 (*r* = 0.00)	0.257 ( $\eta_{\varepsilon }^{2}=0.01$)
	Low	53	-0.50 [-0.80, -0.30]	< 0.001 (*r* = 0.57)	
	Moderate	40	-0.80 [-1.20, -0.45]	< 0.001 (*r* = 0.72)	
	High	3	-0.70 [-2.80, -0.30]	0.250 (*r* = 0.66)	
*Education*	Pre-school	6	+0.65 [-0.60, +1.70]	0.438 (*r* = 0.32)	0.024 ( $\eta_{\varepsilon }^{2}=0.07$)
	Poor	4	-0.45 [-3.20, -0.30]	0.125 (*r* = 0.77)	
	Moderate	62	-0.55 [-0.90, -0.35]	< 0.001 (*r* = 0.63)	
	Excellent	28	-0.70 [-1.10, -0.50]	< 0.001 (*r* = 0.82)	
*Family History*	Negative	40	-0.85 [-1.20, -0.40]	< 0.001 (*r* = 0.73)	0.189 (*r_rb_* = 0.16)
	Positive	60	-0.50 [-0.70, -0.30]	< 0.001 (*r* = 0.55)	
*Monitoring*	No	16	-0.65 [-1.20, -0.50]	< 0.001 (*r* = 0.88)	0.364 (*r_rb_* = 0.14)
	Yes	84	-0.55 [-0.85, -0.35]	< 0.001 (*r* = 0.58)	

Within-group analyses utilized Wilcoxon signed-rank tests (r); Between-group analyses utilized Mann-Whitney U (r_rb_) for 2 groups and Kruskal-Wallis H ( $\eta_{\varepsilon }^{2}$) for ≥3 groups.

#### 3.5.2 Knowledge gain by subgroup

Analyses of total knowledge score improvements demonstrated positive score gains across all evaluated subgroups immediately post-intervention, with further retention or marginal increases observed at the 4-month follow-up point. Patients in the high SES category (n = 3) recorded the highest absolute numeric point gains (Median = +13.00 at 4 months), followed by patients with “Good” baseline HbA1c control (Median = +7.00) (**Table 6**).

**Table 6:** Median total knowledge score gain by subgroup.

*Subgroup*	*Category*	*N*	*Median gain [IQR] (post-intervention)*	*Median gain [IQR] (4-month follow-up)*
*Sex*	*Male*	*42*	*+6.00 [5.00, 7.00]*	*+6.00 [5.00, 7.50]*
	*Female*	*58*	*+5.00 [4.50, 5.00]*	*+5.00 [4.00, 7.00]*
*Age Group*	*Toddler/Pre*	*9*	*+7.00 [5.00, 10.00]*	*+6.50 [4.00, 8.00]*
	*School*	*45*	*+5.00 [5.00, 6.00]*	*+5.00 [4.00, 6.00]*
	*Adolescent*	*46*	*+5.00 [4.50, 6.00]*	*+7.00 [5.00, 8.00]*
*Baseline HbA1c*	*Good (*≤*7.5%)*	*19*	*+6.00 [4.00, 7.00]*	*+7.00 [4.00, 10.00]*
	*Fair (7.6–9.9%)*	*40*	*+5.00 [4.00, 7.00]*	*+5.00 [4.00, 6.00]*
	*Poor (*≥*10%)*	*41*	*+5.00 [5.00, 6.00]*	*+6.00 [5.00, 8.00]*
*SES*	*Very Low*	*4*	*+6.50 [5.00, 10.00]*	*+7.50 [5.00, 11.00]*
	*Low*	*53*	*+5.00 [4.00, 6.00]*	*+5.00 [4.00, 7.00]*
	*Moderate*	*40*	*+5.00 [5.00, 6.50]*	*+6.00 [4.00, 7.00]*
	*High*	*3*	*+9.00 [8.00, 11.00]*	*+13.00 [6.00, 17.00]*
*Education*	*Pre-school*	*6*	*+6.00 [2.00, 8.50]*	*+6.50 [2.50, 10.00]*
	*Poor*	*4*	*+5.50 [1.00, 13.00]*	*+3.50 [1.00, 20.00]*
	*Moderate*	*62*	*+5.00 [5.00, 6.00]*	*+5.50 [4.50, 7.00]*
	*Excellent*	*28*	*+5.00 [4.50, 6.50]*	*+6.00 [5.00, 7.00]*
*Family History*	*Negative*	*40*	*+5.00 [5.00, 6.00]*	*+6.00 [5.00, 8.00]*
	*Positive*	*60*	*+5.00 [4.50, 6.00]*	*+5.50 [4.00, 7.00]*
*Monitoring*	*No*	*16*	*+5.00 [3.50, 5.00]*	*+5.00 [3.00, 6.00]*
	*Yes*	*84*	*+5.00 [5.00, 6.00]*	*+6.00 [5.00, 7.00]*

### 3.6 Complications, hospital admissions, and quality of life

Despite the young age of the cohort, microvascular complications were alarmingly prevalent at baseline (23.0% overall), primarily driven by neuropathy (18.0%), followed by retinopathy (9.0%) and nephropathy (5.0%). Logistic regression confirmed that baseline HbA1c was the dominant risk factor, with each 1% increase in HbA1c raising the odds of any complication by 63.4% (OR = 1.634, 95% CI: 1.18–2.25, p = 0.002). Furthermore, patients engaged in regular glucose monitoring exhibited a significantly lower prevalence of retinopathy (Fisher’s Exact p = 0.034), underscoring the protective effect of disease surveillance.

**Figure 5:**
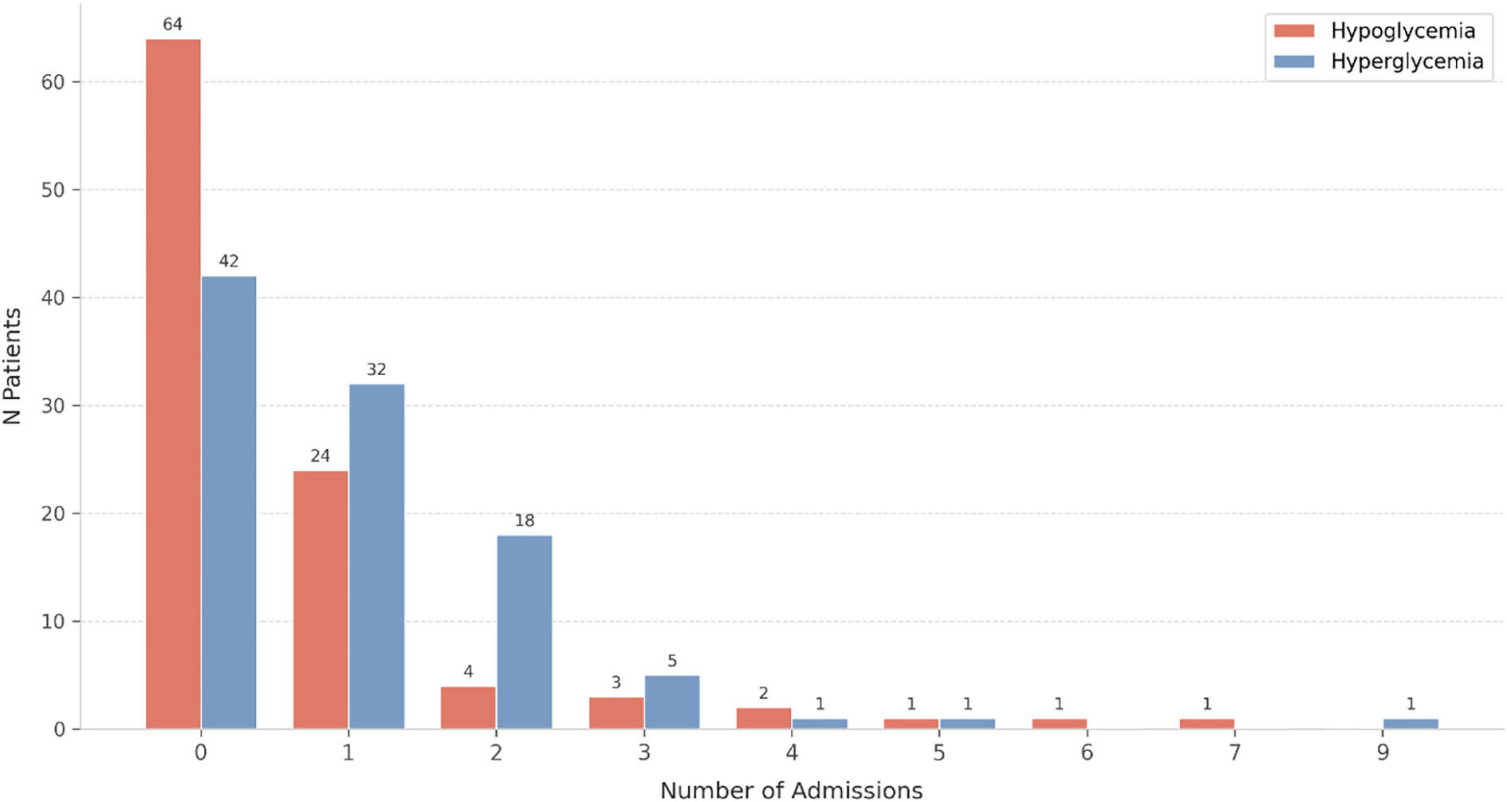
Hyperglycemia and hypoglycemia prior to hospital admissions.

Historically, 60.0% of the cohort had experienced prior hospital admissions for hyperglycemic crises, and 35.0% for severe hypoglycemia. Remarkably, zero hospital admissions were recorded during the 4-month study period. While the lack of a control group necessitates cautious interpretation, this complete cessation of acute crises strongly suggests that the combined effects of the educational intervention, enhanced family vigilance, and telemedicine follow-ups successfully mitigated acute glycemic derangements (**Figure 5**).

Quality of Life (QoL), as measured by the PedsQL, revealed that most children felt their diabetes “Almost Never” or “Sometimes” interfered with daily activities. However, worse glycemic control (higher baseline HbA1c; *r* = 0.220 for Total QoL) and a history of hypoglycemic admissions (*r* = 0.222 for Total QoL) or hyperglycemic hospital admissions (*r* = 0.352 for Total QoL) were significantly correlated with worse QoL scores across multiple domains (all *p* < 0.05).

### 3.7 Reliability and internal consistency

The diabetes self-management knowledge questionnaire demonstrated acceptable to good internal consistency across assessment time points. Cronbach’s alpha was 0.683 at baseline and improved to 0.771, 0.768, and 0.777 at the immediate post-intervention, 2-month, and 4-month follow-up assessments, respectively. The Pediatric Quality of Life Inventory (PedsQL) showed good internal consistency (Cronbach’s α = 0.800). Test–retest reliability analysis demonstrated good stability of knowledge scores over time, with an intraclass correlation coefficient (ICC) of 0.804.

## 4 Discussion

This study evaluated the effectiveness of a structured diabetes self-management education program incorporating high-definition video technology in improving diabetes-related knowledge and glycemic control among children with T1D.

The program was conducted by a diabetes specialist and a dietician with special experience in the nutrition of diabetes. The efficacy of diabetes education was evaluated by using a questionnaire that covered all aspects of the educational program, including general knowledge about diabetes, complications as hypo and hyperglycemia, insulin injection, diabetic nutrition and psychological support, and follow-up awareness.

A key strength of the educational program was the individualized carbohydrate counting provided by a trained dietitian, which allowed dietary recommendations to be tailored to each patient’s age, insulin regimen, and daily activity levels. This personalized approach, coupled with follow-up assessments at 2 and 4 months, supported sustained engagement and reinforced self-management behaviors, ultimately contributing to improved metabolic outcomes.

The observed reduction in HbA1c following the educational intervention may reflect improved adherence to insulin therapy, dietary management, and self-monitoring of blood glucose, which are key components of diabetes self-management education. Importantly, the present study demonstrated a significant and independent association between improvement in diabetes-related knowledge and reduction in HbA1c levels. This relationship was confirmed through both correlation and multivariate regression analyses, suggesting that knowledge acquisition may play a mechanistic role in improving glycemic control.

Notably, no hospital admissions for acute glycemic events were recorded during the follow-up period. Although the absence of a control group warrants cautious interpretation, this finding may reflect the combined effect of structured education and telemedicine follow-up in enhancing early recognition and management of acute complications.

Importantly, the educational intervention was effective across diverse sociodemographic backgrounds and was not influenced by age, sex, diabetes duration, family history, or socioeconomic status. This suggests that video-based self-management education represents an inclusive and scalable strategy that can be implemented in resource-limited settings without exacerbating health disparities.

However, subgroup analyses indicated that the intervention was less effective among younger children (toddler/preschool age) and those in the lowest socioeconomic and educational strata. This may be attributed to developmental limitations affecting comprehension in younger children, as well as barriers related to health literacy and resource accessibility in disadvantaged groups. These findings highlight the need for tailored educational strategies for these vulnerable populations.

The low prevalence of chronic diabetes-related complications observed in the study population is consistent with their relatively young age and supports the importance of early educational interventions aimed at preventing long-term disease sequelae through improved metabolic control.

These findings are consistent with those reported by Romero-Castillo et al., who demonstrated that structured diabetes self-management education programs can significantly improve glycemic outcomes (14). The similarity between the two studies may be attributed to the structured nature of the educational interventions and their emphasis on patient engagement and self-management behaviors.

Additionally, the purpose of our thesis was associated with the massive open online courses (MOOCs), which were held over 2 days and a 3‐month follow‐up in 2021 with T1D patients by the development of MyWay Digital Health Ltd./NHS Diabetes Program, and it was approved by the Quality Institute for Self‐Management Education and Training. The outcomes resulting from MOOCs showed that the effective role of the educational part in improving self‐management in T1D (15).

The consequence of our research may agree with Mauri A et al., 2017, who developed a pediatric education program and confirmed the effectiveness in altering the three levels of “knowing”, “know-how”, and “well-being” required to optimize the overall well-being of young patients with T1D (16).

Similar findings have been reported in several previous studies. Alonso et al. demonstrated that diabetes education improves metabolic control among pediatric patients with type 1 diabetes (17).

Likewise, Beck et al. found that higher diabetes knowledge scores were associated with lower HbA1c levels, highlighting the importance of patient and caregiver education (18). Additionally, Assaad et al. reported significant reductions in HbA1c levels following educational interventions aimed at improving diabetes-related knowledge and self-management practices (19).

As well as the results above, the findings of the present study were supported by Burnett SM, 2017 findings who studied the effect of nutrition and diabetes education programs on improving A1C knowledge and A1C blood levels, and it showed that diabetes education and nutrition programs greatly increased blood A1C levels and awareness of A1C. These results implied that diabetes education and nutrition initiatives were helping older persons better control their diabetes (20).

Lastly, this finding was similar to the results of the study carried out by Davis RM et al., 2010, which studied the effect of using Tele Health in improving diabetes self-management in an underserved Community and reported that there was a significant reduction in glycated hemoglobin in the Diabetes Tele care group from baseline to 6 and 12 months compared with usual care (21).

### 4.1 Strengths

This study has several strengths. First, it focuses on a clinically important and vulnerable population of children and adolescents with type 1 diabetes, a group that requires continuous education and support for effective disease management. The content of the intervention, an interactive high-definition video, was combined with individualized dietary counseling, including carbohydrate counting provided by a trained dietitian, along with scheduled follow-up at 2 and 4 months, reflecting a practical multidisciplinary approach to diabetes education.

Second, the study evaluated both objective and subjective outcomes. In addition to the objective and widely accepted marker of glycemic control, namely HbA1c%, structured instruments were used to assess diabetes-related knowledge, socioeconomic status, and quality of life. Repeated assessment of knowledge at baseline, 2 months, and 4 months enabled evaluation of both short-term improvement and early retention of diabetes education.

Third, the educational strategy was designed to be applicable in resource-constrained settings. The use of video-based education, oral questionnaire administration for caregivers with limited literacy, and telemedicine follow-up through phone calls and SMS suggests that this model could be adapted and implemented in similar low- and middle-income settings. Finally, the intervention appeared to be effective across different sociodemographic groups, supporting its potential applicability in socially diverse populations.

Additionally, the study provides mechanistic insight by demonstrating an independent association between knowledge gain and glycemic improvement through regression analysis.

### 4.2 Limitations

Several limitations, however, need to be acknowledged. Since it was a single-arm pre–post design without a control group, the improvement observed cannot solely be attributed to the educational intervention. Increased clinical contact, regression to the mean, or concurrent changes in care could also have contributed to HbA1c reduction and improvement in knowledge scores.

This sample was recruited by convenience from two outpatient clinics and thus cannot fully represent all children and adolescents with T1D in other regions or health-care settings. The relatively modest sample size also reduces statistical power to detect subgroup differences and thus may account for not finding significant associations between knowledge improvement and sociodemographic variables.

Moreover, the follow-up period was relatively short. HbA1c was reassessed at 4 months, and knowledge was evaluated up to 4 months after the intervention. Thus, conclusions about the long-term sustainability of the improvements observed or their impact on the incidence of acute or chronic complications are precluded. Behavioral outcomes like adherence to insulin therapy, frequency of self-monitoring of blood glucose, and dietary adherence were not systematically measured, limiting insight into mechanisms underlying the improvement in HbA1c. Another limitation is the use of a questionnaire to assess knowledge, partly in an oral form, which may introduce information and social desirability bias, particularly among caregivers with low literacy levels. In addition, although validated tools were used for the assessment of socioeconomic status and quality of life, the diabetes knowledge questionnaire itself was not reported to be a previously validated instrument, which may affect the generalizability and comparability of the findings. Furthermore, quality of life was assessed only at baseline and was not evaluated longitudinally, limiting conclusions regarding the impact of the intervention on patient-reported outcomes.

### 4.3 Recommendations

The review’s data indicates that this education program should be implemented frequently, at least once a month, during patients’ routine maintenance checkups at the diabetes outpatient clinic.

Since nutrition therapy plays a big role in managing diabetes, more study is required in this field. For instance, various teaching styles may be more effective than others at enhancing knowledge, practice, and metabolic regulation when it comes to presenting nutritional information.

Longer-term cohort studies and trials with longer follow-up will be necessary to evaluate the impact of education on the long-term consequences of diabetes because the disease’s complications build over many years. To encourage parents to use and renew their knowledge on diabetic nutrition, CHO counting sessions must be attended multiple times. Noting the findings of the most recent investigations in the patient files.

To reduce DKA as the initial presentation, the media should raise awareness of T1D; this should include a high index of suspicion for both doctors and the general public.

## 5 Conclusion

This study demonstrated that a structured diabetes self-management education program incorporating high-definition video technology was associated with significant improvements in children’s understanding of T1D and its management, alongside a significant reduction in HbA1c levels. Importantly, knowledge gain was independently associated with glycemic improvement, highlighting a potential mechanistic role of structured education in clinical outcomes.

The intervention appeared effective across diverse sociodemographic groups, supporting its scalability in resource-limited settings. However, variability in response suggests that tailored approaches may be needed for younger children and those from lower socioeconomic and educational backgrounds.

Overall, video-based diabetes education combined with structured follow-up represents a practical and effective strategy to improve diabetes knowledge and glycemic control.

## Data Availability

The raw data supporting the conclusions of this article will be made available by the authors, without undue reservation.
